# Proficiency in Using Level Cue for Sound Localization Is Related to the Auditory Cortical Structure in Patients With Single-Sided Deafness

**DOI:** 10.3389/fnins.2021.749824

**Published:** 2021-10-11

**Authors:** Ja Hee Kim, Leeseul Shim, Junghwa Bahng, Hyo-Jeong Lee

**Affiliations:** ^1^Department of Otorhinolaryngology-Head and Neck Surgery, Hallym University College of Medicine, Chuncheon, South Korea; ^2^Laboratory of Brain & Cognitive Sciences for Convergence Medicine, Hallym University College of Medicine, Anyang, South Korea; ^3^Department of Audiology and Speech-Language Pathology, Hallym University of Graduate Studies, Seoul, South Korea

**Keywords:** hearing loss, unilateral hearing loss, sound localization, neuronal plasticity, gray matter volume

## Abstract

Spatial hearing, which largely relies on binaural time/level cues, is a challenge for patients with asymmetric hearing. The degree of the deficit is largely variable, and better sound localization performance is frequently reported. Studies on the compensatory mechanism revealed that monaural level cues and monoaural spectral cues contribute to variable behavior in those patients who lack binaural spatial cues. However, changes in the monaural level cues have not yet been separately investigated. In this study, the use of the level cue in sound localization was measured using stimuli of 1 kHz at a fixed level in patients with single-sided deafness (SSD), the most severe form of asymmetric hearing. The mean absolute error (MAE) was calculated and related to the duration/age onset of SSD. To elucidate the biological correlate of this variable behavior, sound localization ability was compared with the cortical volume of the parcellated auditory cortex. In both SSD patients (*n* = 26) and normal controls with one ear acutely plugged (*n* = 23), localization performance was best on the intact ear side; otherwise, there was wide interindividual variability. In the SSD group, the MAE on the intact ear side was worse than that of the acutely plugged controls, and it deteriorated with longer duration/younger age at SSD onset. On the impaired ear side, MAE improved with longer duration/younger age at SSD onset. Performance asymmetry across lateral hemifields decreased in the SSD group, and the maximum decrease was observed with the most extended duration/youngest age at SSD onset. The decreased functional asymmetry in patients with right SSD was related to greater cortical volumes in the right posterior superior temporal gyrus and the left planum temporale, which are typically involved in auditory spatial processing. The study results suggest that structural plasticity in the auditory cortex is related to behavioral changes in sound localization when utilizing monaural level cues in patients with SSD.

## Introduction

Unilateral loss of hearing deteriorates sound localization ability, which relies largely on binaural time and level differences in the sound source that reaches the two ears. Loss of binaural time/level cues results in poor localization performance, which is a significant behavioral deficit in patients with asymmetric hearing (Kumpik and King, 2019; Snapp and Ausili, 2020). However, reported data also show considerable variability in the degree to which localization accuracy is affected by monaural hearing, suggesting the adaptive contribution of the monaural level cues and monaural pinna cues, which normally play a minor role. For example, relatively good localization has been found in some adults with various degrees of unilateral hearing loss, whereas others cannot localize at all (Slattery and Middlebrooks, 1994; Van Wanrooij, 2004; Shub et al., 2008; Agterberg et al., 2012; Firszt et al., 2017; Pastore et al., 2020). The variable impact of hearing loss on localization ability, even in individuals with unilateral sensorineural single-sided deafness (SSD), indicates that hearing loss in the poorer ear alone does not eradicate the potential to localize sound. Instead, this variability suggests that malleable processes in higher-level structures occur naturally to improve localization accuracy. Deprived of binaural difference cues, the adaptive change in SSD would rely on increased proficiency of using remaining monaural spatial cues. Monaural level cues are primarily used over monaural spectral shape cues (Shub et al., 2008; Pastore et al., 2020). Although inherently ambiguous, the monaural level cue can serve as a valid cue to azimuth because the learned sound will appear louder when presented on the better-hearing side (Agterberg et al., 2012). A sound source located on the impaired hearing side is attenuated by the subject’s head when reaching the hearing ear side. The degree of attenuation varies according to the azimuthal location relative to the intact ear. This level cue by head shadowing would be unreliable if the source level varies frequently, but it serves as an essential localization cue in a familiar environment. Considering the high prevalence of high-frequency hearing loss, considerable patients with asymmetric hearing might rely on monaural level cues rather than the monaural spectral cues based on high-frequency information. Reflecting the high variability of localization performance and the diversity of the audiologic profile, clinical factors that contribute to improved auditory spatial ability have only been reported in a few studies; longer duration of SSD (Liu et al., 2018; Nelson et al., 2019) and younger age at SSD onset (Firszt et al., 2017; Nelson et al., 2019) have been associated with better localization performance.

Although adaptive changes in localization performance have been reported repeatedly, the neural correlates associated with functional changes have not yet been clearly addressed. Different auditory spatial cues are separately processed in the lower brainstem and are integrated in the higher subcortex or above. Across the auditory neural pathway, functional changes that improve localization performance occur at a higher level, where cue integration takes place and enables the reweighting of spatial cues. The auditory cortex plays a crucial role in auditory spectrotemporal analysis, and functional neuroimaging studies (Pavani et al., 2002; Deouell et al., 2007; Ahveninen et al., 2014; Callan et al., 2015) and lesion studies (Zatorre and Penhune, 2001; Zündorf et al., 2014) have indicated that it is essential for sound localization. Functional magnetic resonance imaging (MRI) studies have shown that sound localization processing has a particular activating effect on areas of the posterior auditory cortex, such as the planum temporale (PT) (van der Zwaag et al., 2011; van der Heijden et al., 2018, 2019). While each auditory cortex preferentially responds to sound location in the contralateral hemifield, right hemispheric dominance (Zatorre and Penhune, 2001; Zündorf et al., 2014; Callan et al., 2015) and the role of interhemispheric connection (Briley et al., 2013; Derey et al., 2016) have been reported. The auditory cortex also has a feedback/feedforward connection to the higher-order cortices that is associated with top-down attentional modulation of auditory spatial ability (van der Heijden et al., 2018, 2019). Meanwhile, neuroanatomical characteristics of the auditory cortex, such as the change of gray matter volume (GM volume), have shown to be associated with certain types of hearing loss (Cardin, 2016). For example, structural changes in the gray matter in the auditory cortex have been found for patients with mild-to-moderate hearing loss (Husain et al., 2011), high-frequency hearing loss (Eckert et al., 2012, 2019), and unilateral hearing loss (Yang et al., 2014; Heggdal et al., 2020). Yet, the evidence for the structural changes relating to auditory performance in sound localization is limited.

In the present study, sound localization ability using monaural level cues was evaluated in 26 patients with SSD and compared with those from acute plugged controls (*n* = 23) to examine the behavioral change in auditory spatial ability in the SSD group. In the SSD group, clinical factors were analyzed to determine whether the duration of SSD/age at SSD onset was related to behavioral changes. To examine the biological substrate in the auditory cortex associated with behavioral adaptation, the GM volume was measured in the parcellated regions of the bilateral auditory cortices and compared to the changes in localization performance using monaural level cues in the SSD group.

## Materials and Methods

### Participants

Demographic and clinical information for all subjects is provided in Table 1. Twenty-six patients who had severe to profound unilateral sensorineural hearing loss (M:F = 12:14; age mean ± SD = 42.7 ± 11.4 years old) participated in the study. Fifteen patients had unilateral hearing loss in their left ear (SSD-L), whereas eleven had hearing loss in their right ear (SSD-R). The inclusion criterion was severe to profound hearing loss in one ear [pure-tone average (PTA) ≥ 70 dB HL] and 30 dB HL or better in the other ear (Vincent et al., 2015; Van de Heyning et al., 2016). Pure tone audiometry was performed at the range of frequencies from 125 to 8000 Hz, and the averaged 4-tone thresholds at 500, 1000, 2000, and 4000 Hz produced PTA. Patients were included only when the duration of severe to profound hearing loss was more than 1 month. Causes of unilateral hearing loss were congenital hearing loss, sudden sensorineural hearing loss, and progressive sensorineural hearing loss in 4, 13, and 9 patients, respectively. For those with a history of progressive hearing loss, the onset and duration of hearing loss were defined based on the time when their hearing loss reached the level of severe hearing loss. When analyzing sound localization performance, the two SSD groups were pooled (details are presented in section “Results”) and subdivided into three subgroups according to their duration of SSD (SSD _<__7__y__ea__rs_: 0.17–7 years of SSD, SSD_10__–__29__y__ea__rs_: 10–29 years of SSD, and SSD_43__–__46__y__ea__rs_: 43–46 years of SSD).

**TABLE 1:** Demographic characteristics of the groups.

	SSD-L (*n* = 15)	SSD-R (*n* = 11)	NC (*n* = 23)	*p*-Value
Age (years)	38.9 ± 12.1	46.1 ± 9.1	41.7 ± 11.9	0.929
Sex (M/F)	8/8	4/7	11/12	0.707
Age at onset of SSD (years)	24.3 ± 14.4	24.9 ± 20.0	–	0.755
Duration of SSD (years)	14.5 ± 12.8	21.1 ± 20.2	–	0.716
Good-ear PTA (dB HL)	14.0 ± 12.6	13.8 ± 9.6	10.0 ± 7.2 (Lt)	0.357
Poor-ear PTA (dB HL)	108.2 ± 15.5	104.0 ± 19.3		0.936

*SSD-L and SSD-R denote patient groups with single-sided deafness in the left and right ears, respectively.*

*NC, normal control group; M, male; F, female; dB HL, dB hearing level.*

Twenty-three healthy subjects (M:F = 11:12; age mean ± SD = 41.7 ± 12.0 years) with normal hearing (PTA less than 25 dB HL in both ears) without a history of hearing problems served as the normal control (NC) group. The participants were matched to the SSD group in terms of the hearing threshold in the intact ear, sex, and age.

All participants were right-handed. Subjects were excluded if they had any neurological/neuropsychiatric history or took any related medication. Medical history including diabetes, hypertension, and any condition that contraindicated MRI scanning were also exclusion criteria.

This study protocol was approved by the Institutional Review Board of the Hallym University Sacred Heart Hospital (Anyang, South Korea) (IRB No. 2018-02-019-002).

### Sound Localization Test

**FIGURE 1:**
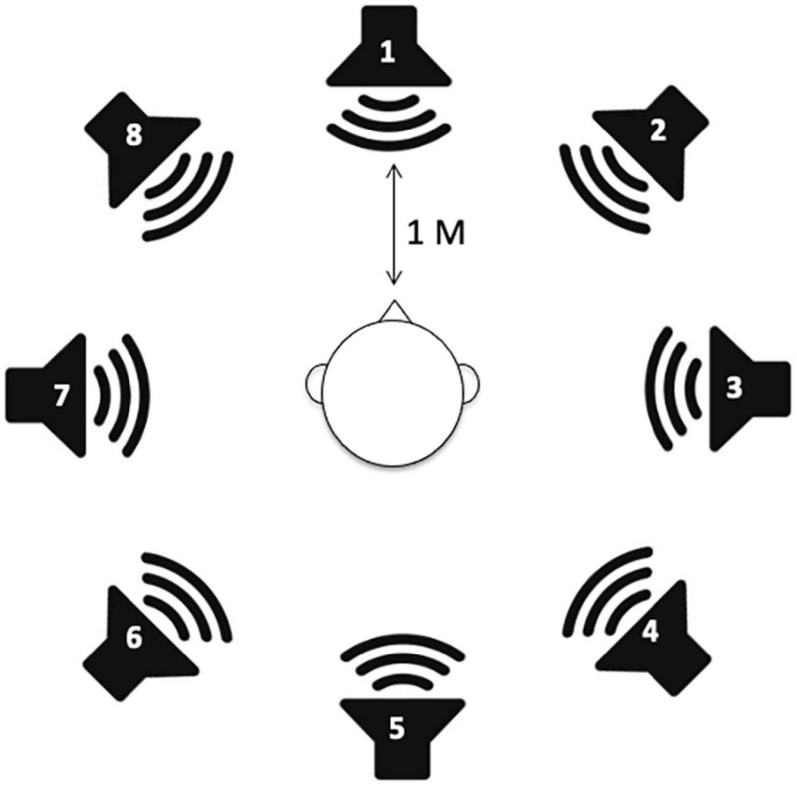
Speaker setup for sound localization measurements. A total of eight speakers are positioned in a full circle with 45° angular spacing.

A sound localization test was performed using a DHA-8 apparatus (Directional Hearing Evaluator 8, Interacoustics, Denmark) that assesses a person’s ability to identify the source location of a sound. Eight speakers were used in the experiment with two speakers in the front and rear of the subject (at 0° and 180°) and three additional speakers on each side of the individual, separated by 45°. All speakers were mounted at head level and labeled no. 1 to no. 8 (Figure 1).

As auditory stimuli, a 1 kHz warble tone at 40 dB SL was presented using GSI 61 Audiometry (Grason-Stadler, MN, United States). The duration of the sound presentation was 3 s, with an approximate 5 s interstimulus interval. Five trials from each of eight loudspeakers were presented in random order, and a total of 40 trials were tested per subject. Presentation^®^ software (Neurobehavioral System, Albany, CA, United States) was used to randomize trials and to record responses.

The subjects were seated in the center of the sound booth and a chair facing the loudspeaker at 0° azimuth (front) and at 1 m from the speakers in any direction. Prior to the test, a training session composed of randomized trials of three presentations per speaker was conducted. Subjects answered the sound source by the number of speakers using their own voice. Participants were asked not to move their head in the course of the experiment. The response angle was averaged for five trials per speaker. To calculate the mean absolute error (MAE), the absolute difference between the response speaker and stimulus speaker was averaged for five trials per speaker, meaning smaller values indicated better sound localization. An asymmetry index (AI) was calculated to reflect performance discrepancies between the good- and poor-hearing sides. AI was defined as a relative difference in weighted angle differences between good- and poor-ear sides using the following equation:

$$\text{AI}=\frac{\begin{array}{c} \left(\mathrm{Poor}-\mathrm{ear}\,\mathrm{speaker}:225^{\circ }+2\times 270^{\circ }+315^{\circ }\right)\\ -\left(\mathrm{Good}-\mathrm{ear}\mathrm{speaker}:45^{\circ }+2\times 90^{\circ }+135^{\circ }\right) \end{array}}{\begin{array}{c} \left(\mathrm{Poor}-\mathrm{ear}\,\mathrm{speaker}:225^{\circ }+2\times 270^{\circ }+315^{\circ }\right)\\ +\left(\mathrm{Good}-\mathrm{ear}\mathrm{speaker}:45^{\circ }+2\times 90^{\circ }+135^{\circ }\right) \end{array}}$$

This index may span from −1 to 1. When this index approaches zero, it means that the sound localization performance is similar across the good- vs. poor-hearing ear sides. When the good-hearing side markedly outperformed the poor-hearing side, the AI was approximately 1.

The SSD group was tested *via* the intact ear. In the NC group, a simulation experiment was conducted to compare the results with those of the SSD group. The right ear was covered with an earmuff and subjected to narrowband noise (centered at 1 kHz) for masking using an insert earphone (ER3-A, Etymotic Research Inc. Elk grove village, IL, United States), which prevented auditory stimuli from being processed by the covered right ear. The narrowband noise for the masking was provided through an audiometer at 20 dB SL for frequencies above 1 kHz (AudioStar Pro; GSI Grason-Stadler, Eden Prairie, MN, United States).

### Structural Analysis of the Auditory Cortex

#### Structural Magnetic Resonance Imaging

High-resolution 3D-T1 weighted images were obtained using a 3T MRI scanner (Philips, Amsterdam, Netherlands) in both the SSD and NC groups. A 3D-gradient fast field echo sequence was applied with repetition time (TR) = 9.3 ms, echo time (TE) = 4.6 ms, field angle of field of view (FOV) = 230 × 230 mm, and matrix size = 230 × 200. The slice thickness was 1.0 mm, for a total of 160 slices without gaps.

#### Image Preprocessing

The cortical volume of the gray matter was generated using voxel-based morphometry (VBM) with the Dartel toolbox running within SPM8 (Statistical Parametric Mapping^1^) implemented in MATLAB version R2009a (MathWorks Inc., Natick, MA, United States) (Ashburner and Friston, 2000; Good et al., 2001). First, the 3-D T1 image of each participant was segmented into gray matter, white matter, and cerebrospinal fluid using a unified tissue segmentation procedure after image intensity nonuniformity correction. Flow fields and a series of template images were generated and used to modulate the spatially normalized brain volume to Montreal Neurological Institute (MNI) space by nonaffine warping. Eventually, a modulated gray matter image was obtained for each subject to detect differences in cortical volume.

#### Normalized Cortical Volume in the Parcellated Auditory Cortex

Three regions of interest (ROIs) per hemisphere (a total of six ROIs) were extracted from the Harvard-Oxford cortical and subcortical structural atlases using the FSL anatomy toolbox.^2^ The ROIs encompassing auditory regions were the anterior part of the superior temporal gyrus (STG), posterior part of the STG, and PT. These ROIs were binarized and registered to the individual’s anatomical space. Additionally, a gray matter mask was applied to transformed ROIs to extract distinct anatomical indices. Finally, to correct for variation in brain size across the individuals, normalized cortical volume (nCV), which represents the GM volume of the predefined ROIs divided by whole-brain volume, was calculated for each ROI.

### Statistical Analysis

Statistical analyses were conducted with R using the ppcor, jmv, dunn.test, and WRS2 packages (Kim, 2015; Mair and Wilcox, 2020; Selker et al., 2020). A normality test was performed using Shapiro–Wilk’s test. Group differences in age and sex distribution were tested using one-way ANOVA and Fisher’s exact test, respectively. In the NC group, PTA across the right and left ears was compared using a paired *t*-test. Between the two SSD groups, the age at SSD onset, the duration of SSD, and PTA in the poor-hearing ear were compared using the Wilcoxon rank sum test. The PTA in the good-hearing ear was compared across the two SSD groups and the acutely plugged NC group (left ear) using the Kruskal–Wallis test.

To analyze sound localization test results, response angles and MAE were analyzed to test the group differences and speaker directions effect (with interaction), using robust ANOVA for mixed design provided by WRS2 package. Nonparametric repeated-measures ANOVA tested the stimulation direction effect on the MAE across groups. The Wilcoxon rank sum test was used to compare the AI values and the MAE in each direction between the NC and SSD groups. For comparison across NC and SSD subgroups, the Kruskal–Wallis test was performed with Dunn’s *post hoc* test with the *p*-value adjusted by the Benjamini–Hochberg method. Nonparametric partial correlation analyses were carried out between sound localization performances (MAE and AI) and clinical factors (age at SSD onset and duration of SSD), controlling for age.

When analyzing cortical structure, two SSD groups were analyzed separately, considering the innate structural/functional asymmetry of the brain. The nCV in each ROI was compared across groups using robust one-way ANOVA based on trimmed means. A nonparametric partial correlation was used to examine whether the nCV extracted from six ROIs was related to the age at onset of SSD, duration of SSD, and AI, controlling for age at the time of the experiment.

## Results

### Demographic Characteristics

Across the three groups, age [*F*(2,46) = 1.273, *p* = 0.290] and sex distribution (Fisher’s exact test, *p* = 0.707) were not significantly different. PTA in the good (tested) ear was also not different across the three groups [χ^2^(2) = 2.062, *p* = 0.357]. Between the two SSD groups (SSD-R vs. SSD-L), there were no significant differences in PTA in the poor ear (Wilcoxon rank sum test, *p* = 0.936), duration of SSD (*p* = 0.716) or age at onset of SSD (*p* = 0.755).

When the two SSD groups were pooled, the duration of SSD was negatively correlated with the age at onset of SSD (*r_*s*_* = −0.793, *n* = 26, *p* < 0.001), but the age at the time of the experiment was related to neither the duration (*p* = 0.271) nor the age at onset of SSD (*p* = 0.101). When the two SSD groups were separately tested, a negative correlation between age at onset and duration of SSD was significant in both SSD groups (SSD-R: *r*_*s*_ = −0.834, *p* = 0.001; SSD-L: *r*_*s*_ = −0.606, *p* = 0.017). The age at the time of the experiment was significantly related to the age at onset of SSD in the SSD-L group only (SSD-R: *p* = 0.422; SSD-L: *r*_*s*_ = 0.581, *p* = 0.023) but was not related to the duration of SSD in either group (SSD-R: *p* = 0.705, SSD-L: *p* = 0.334).

In the NC group, PTA from both ears was not different [*t*(22) = 1.464, *p* = 0.157].

### Behavioral Results

When the localization test results of the SSD-L group were flipped to match the good-/poor-hearing side with those of the SSD-R group, the response angle was different across stimulation directions, whereas no differences were found for groups. A mixed design ANOVA results showed no significant interaction between stimulation direction and group [robust ANOVA for mixed design; stimulation direction: *F*(7,11.1) = 26.8, *p* < 0.001; group: *F*(1,13.7) = 0.6, *p* = 0.447; stimulation direction × group: *F*(7,11.1) = 1.42, *p* = 0.288]. Therefore, all data reported here were standardized such that negative angular or stimulus values represent the impaired (plugged) ear side and positive values represent the intact (open) ear side. The pooled data from the SSD group were compared with those from the NC group (the right ear was covered and masked to simulate SSD-R).

#### Sound Localization Performance

**FIGURE 2:**
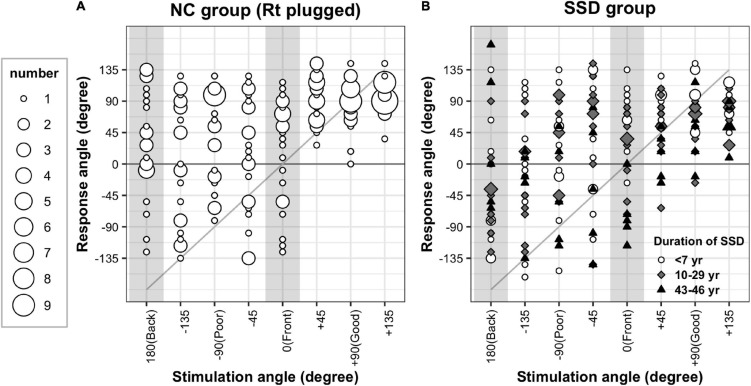
Sound localization test results. Response angles were plotted by stimulation angle in the acutely plugged NC **(A)** and SSD **(B)** groups. Both groups showed response bias toward the intact (open) ear side. Upon all responses, 68.2 and 67.3% were recorded on the intact ear side in the simulated NC and SSD groups, respectively.

The sound localization performance of all individuals in the NC and SSD groups is provided in Figure 2. The results of the simulated NC group revealed that although all responses from the intact ear side (+45°, +90°, and +135°) were on the same hemifield (Figure 2A), the responses from the acutely plugged ear were more diverse. The SSD patients variably responded to stimuli from the good-hearing sides, and some patients even responded to the opposite hemifield with stimuli from the good-hearing side (Figure 2B). A robust ANOVA for mixed design was examined the effect of group and stimulation angles on behavioral performances. Results showed a significant effect of the stimulation angles, with no effects for the group and interaction [stimulation direction: *F*(7,19.9) = 13.7, *p* < 0.001; group: *F*(1,19.8) = 3.2, *p* = 0.087; stimulation direction × group: *F*(7,19.9) = 1.98, *p* = 0.109]. When the SSD group was divided into three subgroups by the duration of SSD (Figure 2B), stimulation angle and its interaction with subgroup showed significant effects, whereas group differences did not [robust ANOVA for mixed design; stimulation direction: *F*(7,3.9) = 19.3, *p* = 0.007; subgroup: *F*(2,3.9) = 5.27, *p* = 0.078; stimulation direction × subgroup: *F*(14,3.8) = 14.3, *p* = 0.012].

Both the NC and SSD groups showed a clear response bias toward the intact side. While 37.5% of the stimuli were provided on the good-hearing side (three out of eight speakers), 68.2 and 67.3% of responses were recorded on this hemifield in the plugged NC and SSD groups, respectively. However, in the impaired side of the SSD subgroup with a very long duration of SSD (43–46 years), bias toward the intact ear side was not apparent, of whom three out of five responded with the correct hemifield to stimuli from three speakers on the poor-hearing side (Figure 2B, black triangles).

**FIGURE 3:**
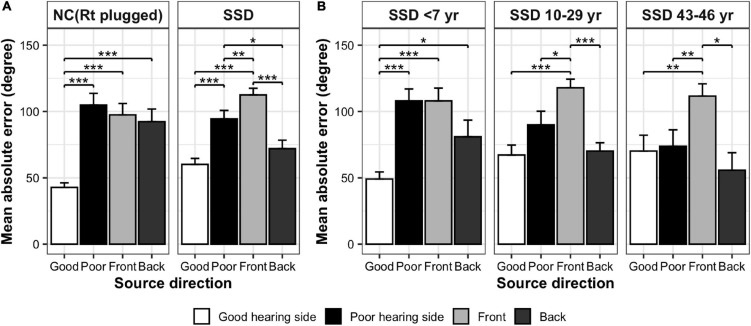
Comparison of MAE values in four directions (mean ± SE). **(A)** NC (acutely plugged) group and SSD group. **(B)** SSD subgroups with different duration of SSD. ***p < 0.001; **p < 0.01; *p < 0.05.

Mean absolute error values between the stimulation angle and response angle were grouped in four directions (front, back, good-ear side, and poor-ear side) and compared between groups (Table 2 and Figure 3). When comparing the NC and SSD groups, the MAE values in the four directions were significantly different across stimulation angles, and interaction was significant; however the group effect was not significant [robust ANOVA for mixed design; group: *F*(1,24.3) = 0, *p* = 0.967; stimulation direction: *F*(3,21.3) = 41.3, *p* < 0.001; stimulation direction × group: *F*(3,21.3) = 3.7, *p* = 0.028]. When comparing each direction separately, the simulated NC group showed significantly better performance (smaller angle difference) than the SSD group for the good-hearing side only (Table 2). On the other three directions, the performances were not significant among groups (Wilcoxon rank sum test; good-hearing side: *p* = 0.008; poor-hearing side: *p* = 0.196; front: *p* = 0.212; and back: *p* = 0.150). In the NC group, MAE values were significantly different across source directions [χ^2^(3) = 25.6, *p* < 0.001, Figure 3A, left panel], with a smaller error on the good-hearing side than on all three other sides (*p* < 0.001). In the SSD group (Figure 3A, right panel), MAE values were also significantly different across directions [χ^2^(3) = 28.6, *p* < 0.001]; the MAE for the good-hearing side was significantly better than that for the poor (*p* = 0.001) and front (*p* < 0.001) directions but was not better than that for the back direction (*p* = 0.234), for which the MAE was also smaller (better) than that for the poor-hearing (*p* = 0.036) and front (*p* < 0.001) directions. When MAE values were further analyzed across the NC group and three SSD subgroups with different durations of SSD (Figure 3B), MAE values were significantly different across source directions [*F*(3,41.9) = 43.7, *p* < 0.001] and interacted with the group [*F*(9,33.6) = 5.4, *p* < 0.001], but the group effect was not significant [*F*(3,30.0) = 0.2, *p* = 0.875]. In the *post hoc* comparison, the group difference was significant only in the good-hearing direction [χ^2^(3) = 10.4, *p* = 0.015], with a significantly smaller MAE in the NC group over two SSD subgroups with over 10 years of SSD (NC vs. SSD_10__–__29__years_: *p* = 0.018, NC vs. SSD_43__–__46__years_: *p* = 0.035). Similar to the NC group, the SSD _<__7__years_ group showed the best MAE on the good-hearing side compared with all three other directions [χ^2^(3) = 13.6, *p* = 0.004; good vs. poor: *p* < 0.001; good vs. front: *p* < 0.001; good vs. back: *p* = 0.048], but in the other two subgroups with over 10 years of SSD, MAE values on the good-hearing side were not significantly superior to the MAE values on the poor-hearing side and back (*p* > 0.05) (Figure 3B).

**TABLE 2:** Mean absolute error values in four directions and asymmetry index values in the SSD and NC groups (mean ± SD).

	Mean absolute error (|stimulus – response|, degree)				AI
Group	Good-hearing side (45°, 90°, 135°)	Poor-hearing side (−45°, −90°, −135°)	Front (0°)	Back (180°)	
NC (left only, *n* = 23)	42.8 ± 16.6	105.0 ± 42.3	97.4 ± 41.4	92.3 ± 45.7	0.42 ± 0.28
SSD (*n* = 26)	60.1 ± 23.1**	94.5 ± 32.1	112.0 ± 25.5	72.0 ± 32.5	0.26 ± 0.30*
SSD_<__7__years_ (*n* = 11)	49.1 ± 17.7	108.0 ± 29.9	108.0 ± 31.9	81.0 ± 41.4	0.41 ± 0.24
SSD_10__–__29__years_ (*n* = 10)	67.2 ± 23.6*	90.0 ± 32.4	118.0 ± 20.5	70.2 ± 19.8	0.20 ± 0.35
SSD_43__–__46__years_ (*n* = 5)	70.2 ± 26.8*	73.8 ± 27.7	112.0 ± 20.7	55.8 ± 29.4	0.02 ± 0.14*

*SSD, single-sided deafness; NC, normal control; AI, asymmetry index.*

The symbols “*” and “**” denote statistically significant differences (*p* < 0.05 and *p* < 0.01, respectively) from the NC group.

The AI values were calculated in each group to represent the difference in sound localization performance between good- and poor-hearing sides (Table 2). AI values were significantly smaller in the SSD group than in the NC group (Wilcoxon rank sum test, *p* = 0.046), meaning that localization performance between the good- and poor-hearing sides was more symmetric in the SSD group than in the acutely plugged controls. When further compared between the NC group and SSD subgroups, AI values differed significantly across groups [Kruskal–Wallis χ^2^(3) = 9.2, *p* = 0.027], with significantly smaller AI values in the SSD_43__–__46__years_ group than in the NC group (*p* = 0.042) and SSD _<__7__years_ group (*p* = 0.033).

#### Localization Performance and Clinical Factors

**FIGURE 4:**
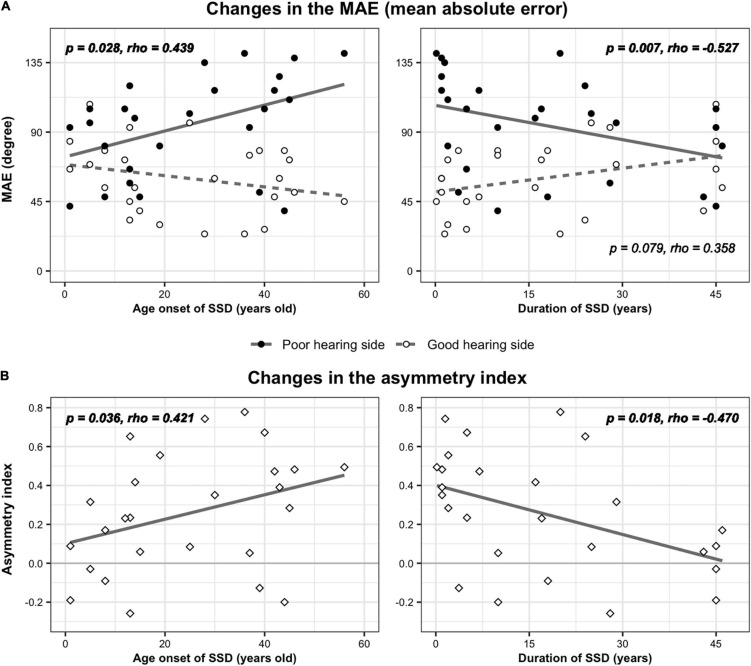
Changes in localization parameters with age of SSD onset and SSD duration. **(A)** MAE values of three speakers on the good- and poor-hearing sides were plotted as a function of onset age (left) and duration (right) of SSD. **(B)** The asymmetry index, which represents the relative difference in weighted MAE values between the good- and poor-hearing sides, was also plotted by the onset age (left) and duration (right) of SSD.

In the SSD group, a partial correlation analysis was performed to examine whether the age at onset or the duration of SSD affected the sound localization performance. While MAE values in the front and back directions were not related to either the duration or age at onset of SSD (*p* > 0.05), MAE values from the poor-hearing side showed a significant correlation with both factors (Figure 4A). In cases of SSD onset at a younger age, localization ability was better (smaller angle difference) on the poor-hearing side (*r*_*s*_ = 0.439, *p* = 0.028). On the good-hearing side, a negative trend showing worse performance with SSD onset at a younger age was found (*p* = 0.124). As expected with the strong negative correlation between onset age and duration of SSD, a reverse relationship was found with the duration of SSD. When the duration of SSD increased, localization performance improved on the poor-hearing side (*r*_*s*_ = −0.527, *p* = 0.007), and tended to deteriorate on the good-hearing side (*r_*s*_* = 0.358, *p* = 0.079). As a result, the difference in sound localization ability between the good- and poor-hearing sides was the largest when the duration of SSD was the shortest (and the age onset of SSD was the latest), and the performance asymmetry between the good- and poor-hearing sides decreased as the duration of SSD increased (and with a younger age of SSD onset). This change in performance asymmetry was confirmed with the analysis of AI in this group. The AI value was smallest when the onset age of SSD was the youngest (*p* = 0.036, *r*_*s*_ = 0.421, Figure 4B, left panel) and the duration of the SSD was the longest (*p* = 0.018, *r*_*s*_ = −0.470, Figure 4B, right panel).

### Auditory Cortex and Asymmetry Index

**FIGURE 5:**
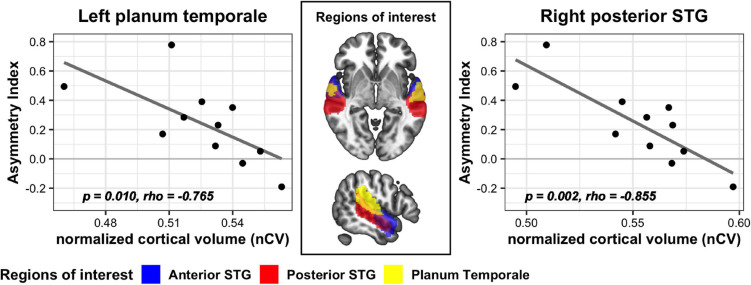
Significant correlation between nCV and AI values in two ROIs of the SSD-R group. STG, superior temporal gyrus.

For analyses of the cortical structural indices, the SSD-R and SSD-L groups were not pooled considering the inherent structural/functional asymmetry of the brain. Across the SSD-R, SSD-L, and NC groups, there was no significant difference in nCV in any of the six ROIs (one-way ANOVA on trimmed means, *p* > 0.05). In both the SSD-R and SSD-L groups, nCV in all six ROIs did not reveal a significant relationship with either the onset age of SSD or duration of SSD (nonparametric partial correlation controlling age, *p* > 0.05). In the SSD-R group, greater volumes in the right posterior STG (*r*_*s*_ = −0.855, *p* = 0.002) and the left PT (*r_*s*_* = −0.765, *p* = 0.010) were associated with smaller AI values (Figure 5), whereas the aging effect was removed. In other words, the greater the GM volume of these regions, the more symmetric the localization performance between the impaired and intact hearing sides in this group. In the SSD-L group and NC group, nCV was not related to AI in any of the six ROIs.

## Discussion

### Auditory Source Localization Using Level Cues Improved on the Deaf Ear Side and Deteriorated on the Intact Ear Side in Single-Sided Deafness Patients

Changes in sound localization behavior in the SSD group observed in the present study can be summarized as follows: (1) In the SSD group, localization ability using level cues on the intact ear side was worse than that in the acutely plugged controls (Table 2) and tended to deteriorate with longer duration of SSD. (2) On the impaired ear side of the SSD group, localization performance improved with longer duration/younger age onset of SSD (Figure 4A). (3) As a result, functional asymmetry between intact and impaired sides decreased in the SSD group, and the maximum decrease was observed with the longest duration/the youngest age onset of SSD (Table 2 and Figure 4B).

For binaural listeners, interaural time and level differences are the two most reliable sources for azimuthal localization, and monaural spectral cues play an auxiliary role, such as discriminating sources in the cone of confusion (Risoud et al., 2018). The monaural spectral cue is generated by the filtering action of the pinna (and head and torso) and composed of high-frequency information. This head-related transfer function is specific to each individual, as the morphology of the body and the pinna varies greatly between individuals. Monaural listeners also heavily rely on monaural level cues, which are generally ignored by binaural listeners (Van Wanrooij, 2004). When the sound source originates from the deaf side, the perceived intensity relates to its azimuth, as the subject’s head attenuates the perceived sound level. Adaptation of this level cue can be measured when the stimulus level is fixed (Van Wanrooij, 2004). Using a 1 kHz sound at a fixed stimulus level, the localization performance measured in this study can reflect the monaural strategy of sound localization using level cues.

On the intact ear side, both the SSD and simulated NC groups showed clear response bias toward the intact (open) ear side (Figure 2), but the MAE values were significantly smaller in the NC group with simulated hearing loss (Table 2). The decreased performance in the SSD group on this side seemed to be associated with SSD experience, a finding supported by the tendency toward increased MAE with increased SSD duration (Figure 4A, right side). The effect of SSD experience was clear on the side with impaired hearing, a finding supported by significant correlations with clinical factors. Their MAE was the best (smallest) when the duration of SSD was the longest or the age at the onset of SSD was the youngest. The AI was calculated by combining the behavioral changes on the intact and impaired sides that changed in opposite ways to represent both factors as a single measure. As expected, Figure 4A shows that the functional asymmetry between the intact and impaired sides decreased with a longer duration/earlier age at the onset of SSD, with a moderate correlation (Figure 4B). A longer duration of SSD (Slattery and Middlebrooks, 1994; Liu et al., 2018), a younger age at the onset of SSD (Firszt et al., 2015, 2017), and both together (Nelson et al., 2019) have been previously associated with improved localization ability in SSD patients. In recent studies that measured localization behavior in SSD patients, those with the best localization performance were those who had more symmetrical abilities on the intact/impaired sides (Agterberg et al., 2012; Nelson et al., 2019). The results of this study replicate those of previous studies showing that auditory spatial strategy changes to help patients cope with single-sided hearing, which attenuates binaural difference cues. However, the magnitude and quality of change varies according to the testing method, the stimuli used, and the audiologic profile of subjects. Behavioral changes are related to the duration and/or onset of unilateral hearing. Overall, the findings suggest adaptive changes in behavior in response to prolonged experience with unilateral hearing and/or developmental plasticity. The demographic profile of our patient cohort did not allow us to differentiate the effects of those two clinical variables. This should be further addressed in future studies with a greater number of patients.

### Gray Matter Volume in the Posterior Part of the Auditory Cortex Is Associated With Auditory Spatial Abilities in Right Single-Sided Deafness Patients

To pursue the biological substrate that grounds the above observed behavioral changes, the cortical structure of the parcellated auditory cortex was compared to the behavioral performance in the SSD group. The AI was used as a composite index representing behavioral changes across impaired and intact sides. Right and left SSDs were separately analyzed considering the functional/structural asymmetry of the auditory cortex. As a result, the GM volume in the right posterior STG and the left PT were strongly related to the AI in the right SSD patients; a greater volume in those areas was related to more symmetric localization performance between the good-/poor-hearing sides.

Auditory spatial processing is mediated by the dorsal stream of the cortical auditory pathway. From the primary auditory cortex, the dorsal stream is directed posteriorly *via* the PT and dorsally to the inferior parietal lobule, premotor cortex, and dorsolateral prefrontal cortex, the latter of which is involved in top-down modulation to the primary and nonprimary auditory regions (van der Heijden et al., 2019). The posterior auditory cortex, including the PT, has been traditionally implicated in spatial processing, especially to the sound source in the contralateral hemisphere (Ahveninen et al., 2014; Callan et al., 2015; van der Heijden et al., 2018).

Compared to bilateral deafness, SSD is a more recent focus of dedicated research, and less is known about neuroplasticity following unilateral hearing loss (Vanderauwera et al., 2020). Although the overall number of studies is small and the audiologic profiles included are diverse, neural changes associated with asymmetric hearing have been observed in both functional and structural domains and are related to clinical/audiologic variables such as the duration of SSD (Yang et al., 2014; Wang et al., 2016), age at SSD onset (Lee et al., 2020), severity of hearing impairment (Wang et al., 2016; Xie et al., 2019), and side (Khosla et al., 2003; Burton et al., 2012; Zhang et al., 2016; Heggdal et al., 2019; Xie et al., 2019; Han et al., 2021). However, findings are scarce regarding how the observed neuroplasticity in asymmetric hearing is related to binaural auditory performance, such as sound localization ability. In one recent fMRI study of subjects with varying degrees of asymmetric hearing loss (Vannson et al., 2020), changes in auditory localization ability were associated with neural plasticity in the posterior auditory cortex, where structural correlation with localization performance was also observed in this study. Neuroimaging studies with sound localization tasks have not yet been reported, and neuroplasticity related to auditory spatial performance changes should be further elucidated with consideration of clinical variables such as the degree, duration, and onset of hearing asymmetry.

This study found cortical structural changes in relation to auditory spatial behavior in the SSD-R group only. Ear-specific neuroplasticity in patients with asymmetric hearing has been reported, yet the findings are controversial. Recent neuroimaging studies have shown a clear impact of the deafness side, with a greater degree of neural plasticity for right-sided hearing loss (Cañete et al., 2019; Heggdal et al., 2020; Han et al., 2021). On the other hand, others reported more extensive neurofunctional reorganization in individuals with left-sided hearing loss (Khosla et al., 2003; Hanss et al., 2009; Zhang et al., 2016). In a series of studies, Zhang et al. (2018a, b) have found different patterns of changes in resting-state connectivity as a function of side of hearing loss. Taken together, previous literature indicates that neuroplastic changes can vary depending on the neural indices applied to measure and the way to examine behavioral performance. In our study, we measured auditory spatial ability, which is known to be dominantly processed in the right hemisphere. Thus, we assumed that in the SSD-R group, auditory input would be efficiently delivered to the right auditory cortex *via* the intact left ear. Accordingly, the relatively preserved sensory input might incur more extensive structural changes in the auditory cortex in this group.

The behavioral changes in auditory spatial ability related to the experience of asymmetric hearing have motivated the development of sound localization training programs and devices for patients with asymmetric hearing loss (Firszt et al., 2015; Fletcher and Zgheib, 2020). A recent study with an animal model reported a specific role of auditory cortical plasticity in sound localization training (Bajo et al., 2019). Improved knowledge regarding the behavioral implications of neuroplasticity in the human auditory cortex would help to improve the effectiveness of those rehabilitative options.

## Conclusion

In SSD patients, sound localization ability changes in relation to the onset/duration of SSD and prolonged experience/earlier onset of SSD were related to a more symmetric performance of using the monaural level cue across the intact and impaired sides. A significant structural correlation of the posterior auditory region to the adaptive behavioral change in sound localization suggests that neuroplasticity occurs in the cortical areas for auditory spatial processing in subjects who suffer from deficits in function due to asymmetric auditory input.

## Data Availability Statement

The raw data supporting the conclusions of this article is available from the corresponding author on reasonable request.

## Ethics Statement

The studies involving human participants were reviewed and approved by the Institutional Review Board of the Hallym University Sacred Heart Hospital. The patients/participants provided their written informed consent to participate in this study.

## Author Contributions

JHK and H-JL contributed to the conception and design of the study. JHK and LS collected and analyzed the data. H-JL and JB contributed to the final version of the manuscript. All authors discussed the result and contributed to writing the manuscript.

## Conflict of Interest

The authors declare that the research was conducted in the absence of any commercial or financial relationships that could be construed as a potential conflict of interest.

## Publisher’s Note

All claims expressed in this article are solely those of the authors and do not necessarily represent those of their affiliated organizations, or those of the publisher, the editors and the reviewers. Any product that may be evaluated in this article, or claim that may be made by its manufacturer, is not guaranteed or endorsed by the publisher.

## Footnotes

1
www.fil.ion.ucl.ac.uk/spm

2
https://fsl.fmrib.ox.ac.uk/fsl/fslwiki/Atlases

## Abbreviations

AI: asymmetry index
GM volume: gray matter volume
MAE: mean absolute error
MRI: magnetic resonance imaging
NC: normal control
nCV: normalized cortical volume
PT: planum temporale
PTA: pure-tone average
SSD: single-sided deafness
STG: superior temporal gyrus.
